# Enhanced Mass Activity and Durability of Bimetallic Pt-Pd Nanoparticles on Sulfated-Zirconia-Doped Graphene Nanoplates for Oxygen Reduction Reaction in Proton Exchange Membrane Fuel Cell Applications

**DOI:** 10.3390/molecules29092129

**Published:** 2024-05-03

**Authors:** Maryam Yaldagard, Michael Arkas

**Affiliations:** 1 Department of Chemical Engineering, Faculty of Engineering, Urmia University, Urmia 5766-151818, Iran; 2 National Centre for Scientific Research “Demokritos”, Institute of Nanoscience and Nanotechnology, 15310 Athens, Greece

**Keywords:** Pt-Pd alloy, sulfated zirconia, graphene nanoplates, cathode, ORR, PEMFC

## Abstract

**Highlights:**

- In this work, graphene nanoplates (GNPs) with a supreme medium were obtained.
- Pt particles (4.50 nm) were uniformly dispersed on the surface of S-ZrO_2_-GNP support.
- The Pt-Pd/S-ZrO_2_-GNPs exhibited higher ECSA than Pt-Pd/ZrO_2_-GNPs and Pt/C.
- Pt-Pd/S-ZrO_2_-GNPs exhibited higher ORR mass activity than other studied electrodes.
- Pt-Pd/S-ZrO_2_-GNPs exhibited low charge transfer resistance in EIS measurements.

**Abstract:**

Developing highly active and durable Pt-based electrocatalysts is crucial for polymer electrolyte membrane fuel cells. This study focuses on the performance of oxygen reduction reaction (ORR) electrocatalysts composed of Pt-Pd alloy nanoparticles on graphene nanoplates (GNPs) anchored with sulfated zirconia nanoparticles. The results of field emission scanning electron microscopy and transmission electron microscopy showed that Pt-Pd and S-ZrO_2_ are well dispersed on the surface of the GNPs. X-ray diffraction revealed that the S-ZrO_2_ and Pt-Pd alloy coexist in the Pt-Pd/S-ZrO_2_-GNP nanocomposites without affecting the crystalline lattice of Pt and the graphitic structure of the GNPs. To evaluate the electrochemical activity and reaction kinetics for ORR, we performed cyclic voltammetry, rotating disc electrode, and EIS experiments in acidic solutions at room temperature. The findings showed that Pt-Pd/S-ZrO_2_-GNPs exhibited a better ORR performance than the Pt-Pd catalyst on the unsulfated ZrO_2_-GNP support and with Pt on S-ZrO_2_-GNPs and commercial Pt/C.

## 1. Introduction

Polymer electrolyte membrane fuel cells (PEMFCs) are increasingly gaining acceptance as a clean, efficient, and silent energy conversion technology, and are seen as a future alternative energy source [1,2,3]. However, the sluggish kinetics of the oxygen reduction reaction (ORR) and the instability of the platinum electrocatalysts for ORR significantly hinder the commercialization of PEMFCs [4,5,6,7]. Accordingly, a higher mass activity and longer durability/stability of the platinum electrocatalysts for ORR are required to increase the popularity of PEMFCs.

It is well understood that the specific activity of the Pt electrocatalyst in the ORR correlates with the carbon support [8]. Carbon supports such as Vulcan have excellent electrical conductivity, chemical stability in acidic solutions, and a large surface area, suitable for the dispersion of catalyst nanoparticles. On the other hand, the latest examinations have shown that the degradation of the electrodes containing carbon supports under the ORR leads to a deterioration in cell efficiency over an extended period [9]. It was found that the electrocatalysts are severely destroyed by the oxidation-induced carbon corrosion under the operating conditions of the cathode, generally due to high potentials, 0.6–1.2 V; high O_2_ concentration; and high temperatures, 50–90 °C. Therefore, the support materials should be more stable to avoid the destruction of the catalyst.

Valve metals, including titanium, zirconium, tantalum, niobium, etc., are known for their high resistance to oxidization as they are passivated in a strongly acidic solution [10]. The oxides of these metals, such as Ti_4_O_7_ [11] and indium tin oxide [12], have been proposed as exceptionally corrosion-resistant materials. These oxides could potentially serve as support materials for cathodes in PEMFCs. In addition, the surfaces of these metal oxides are modified with sulfonic acid (SOx) to act as solid superacids [13]. Specifically, sulfated ZrO_2_ (S–ZrO_2_) is a solid superacid (H0 = −16.03) and is thermally stable at high temperatures of about 500 °C. In their study, Hara and Miyayama discovered that S–ZrO_2_ exhibits high proton conductivity when it has a high atomic ratio of sulfur to zirconia [14]. According to their report, the high proton conductivity was attributed to the localized electrons on the oxygen in SOx and the Lewis acid sites on Zr, which can easily generate new Brønsted acid sites, resulting in a higher conductivity [14]. In the current technology, the addition of a proton conductor, such as a perfluorosulfonated ionomer (PFSI), is crucial for the construction of the wide three-phase boundaries in the back layer of the electrode. The use of a proton-conducting metal oxide as a support for the electrocatalyst would provide an innovative function for the cathode of PEMFCs due to the stability of ZrO_2_. In particular, it is estimated that the amount of altered electrolyte ionomer could be reduced. Reducing ionomer content can improve gas diffusion and water transport. In addition, an increase in the utilization of platinum is expected. Since Pt nanoparticles can not penetrate the tiny pores of the carbon support, they have limited contact with a proton conductor in PFSI [15]. Consequently, the usage of Pt is reduced. S-ZrO_2_ has acidic sites on its surface, even in tiny pores. Therefore, the Pt on S-ZrO_2_ can exchange an H+, which increases the Pt utilization. In addition, the SO4 group on the ZrO_2_ surface increases the hydrophilicity of ZrO_2_ [16,17], which results in S-ZrO_2_ improving the fuel cell performance in low humidity [18]. So because of the solid metal–support interaction, metal oxides improved the ORR activity of the Pt-based catalysts in most examinations [19,20] against the posing effect of the sulfate anion in PFSI in ORR kinetic activity which was recently reported by some authors [21,22,23,24].

Metal oxides generally have poor electrical conductivity. In addition, it is difficult to produce platinum in high concentrations on the metal oxide surface due to the small surface area. Therefore, metal oxides must be modified to increase electrical conductivity or they must be combined with conductive materials such as carbon [25,26]. Designing a complex structure of Pt-based alloys, metal oxides, and carbonaceous materials is a crucial challenge to fully realize their potential. When metal oxides are deposited on carbonaceous materials before the deposition of Pt-based nanoparticles, the Pt-based nanoparticles can deposit on the metal oxides, resulting in poorer ORR activity due to the non-conductivity of the metal oxides. Otherwise, when the Pt-based nanoparticles are first loaded onto the carbonaceous materials, followed by successive deposition of the metal oxide nanoparticles, the metal oxides may cover the Pt-based nanoparticles, resulting in a lower ORR activity because of the reduction in Pt active points. In several studies, metal oxides and carbon were used, but ORR activity could not be increased due to uncontrollable Pt dispersion on metal oxides and carbon [27,28,29]. One approach to solve this problem is to investigate a new carbon-based material as a support.

Over the past few years, graphene—a monolayer of carbon atoms in sp^2^ hybridization arranged in a honeycomb matrix—has attracted considerable technical attention. This unique 2D carbon material exhibits extraordinary properties, such as exceptional electrical, thermal, and mechanical properties [30,31]. As scientists continue to explore its physical and chemical properties, graphene has become one of the most important materials in electronics, nanosensors, nanocomposites, and hydrogen storage [32,33,34]. Due to its large surface area, excellent electrical conductivity, and exceptional mechanical properties, graphene has the potential to serve as a catalyst support in PEMFCs.

The performance of fuel cell electrodes can be improved by using a more active electrocatalyst or by improving the structure of the catalyst layer. In addition to improving the inherent activity of Pt nanoparticles, there is also a strong interest in achieving similar activity improvements by saving Pt to reduce costs. Overcoming the sluggish reduction kinetics of molecular oxygen at low temperatures has been a major challenge in finding an effective catalyst for the cathode side of the fuel cell. Although Pt is generally considered to be the best catalyst for this reaction, successful results have also been obtained with bimetallic Pt alloys [35,36,37,38,39], which exhibit activity towards the ORR that is as good as that of pure Pt in an acidic solution. It is believed that bimetallic Pt alloys improve the durability of PEMFCs by reducing Pt dissolution and migration during operation [39,40,41,42]. Among the many possible combinations of Pt and other metal catalysts, Pd is one of the most commonly used catalysts for ORR in fuel cells operating with acid or alkaline electrolytes [43,44,45,46,47,48]. Moreover, except for Pt, the electrocatalytic activity of Pd due to the electronic properties very similar to Pt is one of the highest among the pure metals for ORR [49,50]. This motivates the development of Pt-Pd alloy catalysts for ORR.

As far as we know, the effectiveness of the platinum–palladium sulfated zirconia/graphene nanoplate (Pt-Pd/S-ZrO_2_-GNP) electrode in the oxygen reduction reaction in PEMFCs has not been investigated. We propose a hybrid electrocatalyst combining sulfated ZrO_2_ nanocrystals with GNP supports and Pt-Pd nanoparticles to achieve better proton and electron conductivity. In the present study, the superacid SO_4_^–2^-ZrO_2_ was synthesized on the GNP surface by chemically linking proton-conducting sulfonic acid groups to form SO_4_^–2^-ZrO_2_-GNP support for Pt-Pd catalysts in PEMFCs. Pt-Pd was dispersed on the surface of S-ZrO_2_-GNPs by the electrodeposition route (Pt-Pd/S-ZrO_2_-GNPs). The purpose for the choice of the electrochemical method was explained in our previous work [51]. The resulting Pt-Pd/S-ZrO_2_-GNP electrode was characterized by physical and electrochemical methods, including AFM, FESEM, TEM, XRD, FTIR, CV, and LSV curves. The study investigated the impact of sulfation on the prepared electrocatalysts (Pt-Pd/S-ZrO_2_-GNPs). The electrocatalysts containing sulfated zirconia oxide–graphene nanoplate (S-ZrO_2_-GNP) powder outperformed the non-sulfated ZrO_2_-GNP powder (Pt-Pd/ZrO_2_-GNP). Additionally, the ORR mechanism behind the role of the S-ZrO_2_ nanoparticles in the electrode structure has been proposed.

## 2. Results and Discussion

### 2.1. Physical Characterization

#### 2.1.1. Topography Study of Graphene Nanoplates and S-ZrO_2_-GNPs

**Figure 1:**
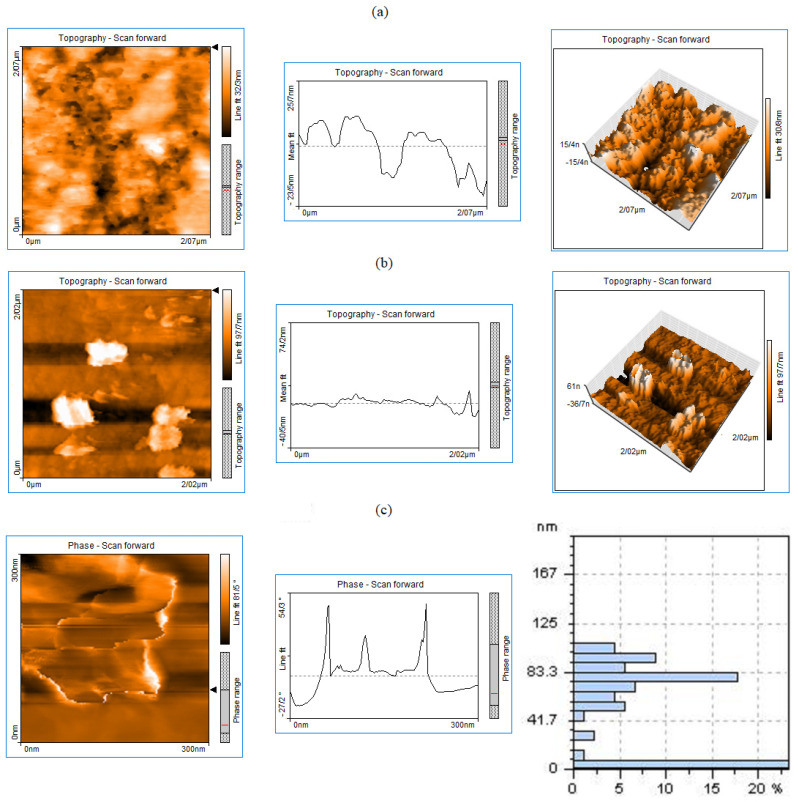
The 2D and 3D AFM topography images along with an equivalent height profile of the (**a**) S-ZrO_2_/GNPs, (**b**) GNPs, and (**c**) the phase scan of GNPs and pore size distribution.

The synthesized graphene nanoplates were studied with atomic force microscopy to specify lateral size and width. Figure 1 shows 2D and 3D AFM images of the ZrO_2_-GNPs with a corresponding height profile along the GNPs derived from the chemical reduction of the exfoliated graphene oxide. The topography height of the graphene nanoplates shows that the graphene nanoplates consist of few layers. As shown in Figure 1c, twenty percent of the produced GNPs have pore size distributions ranging from 1 to 7 nm, while the remaining 80% have a distribution ranging from 8 to 100 nm. Moreover, the images of zirconia nanospheres on graphene nanosheets are visible in Figure 1a. From the assessment (by the Nanosurf Easyscan2 software (Version 2.2.1.16), specific for the used AFM device in this work), the thickness of the S-ZrO_2_-doped graphene nanoplates is about 32–87 nm.

#### 2.1.2. Morphological Study of GNPs and Pt-Pd Nanocrystals on S-ZrO_2_-GNPs

**Figure 2:**
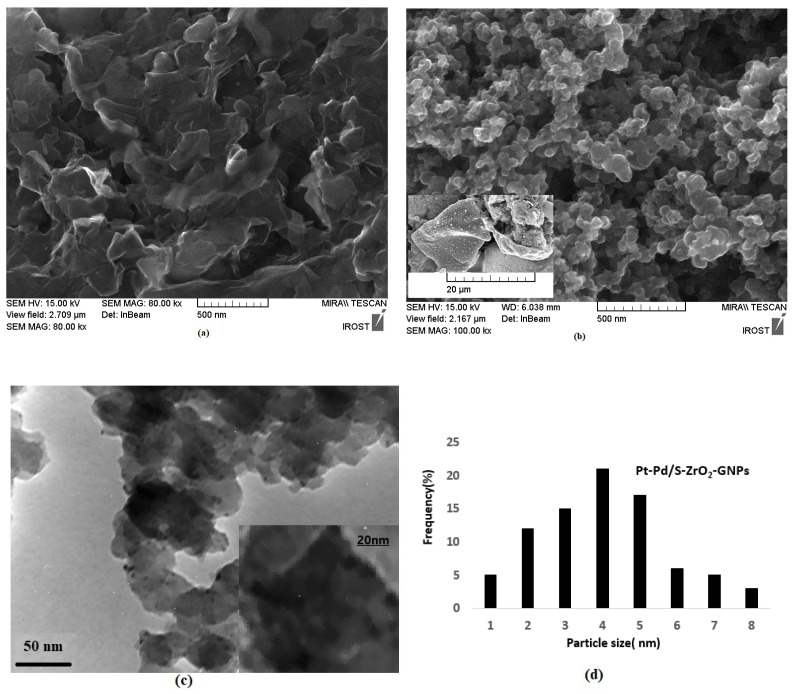
(**a**) FESEM illustrations of graphene nanoplates and (**b**,**c**) FESEM and TEM images of Pt-Pd/S-ZrO_2_-GNPs electrocatalyst. (**d**) Corresponding Pt-Pd nanoparticle size histogram.

Figure 2 shows the general FESEM and TEM illustrations of the (a) GNPs and (b) Pt-Pd/S-ZrO_2_-GNP nanocomposites. Figure 2a reveals a high mass of the chemically prepared GNPs. Figure 2b,c reveal that the uniform spherical Pt-Pd/S-ZrO_2_ nanoparticles were well and homogeneously distributed on the GNPs. Meanwhile, the particle size distribution of Pt-Pd on the S-ZrO_2_–GNPs with sizes ranging from 2 to 8 nm are depicted in Figure 2d. From this histogram, it is concluded that the prepared Pt-Pd nanoparticles were highly dispersed on the support with quite a narrow particle size distribution.

It should be mentioned that to take the microscopic images and peel off the thin layer of the electrodeposit from the surface of the GC electrode, the electrodeposition process was repeated several times. Depending on the number of the repetition cycles of electrodeposition, the Pt-Pd covers the entire surface of the GNPs.

#### 2.1.3. Structural Specifications of Synthesized Support Material

**Figure 3:**
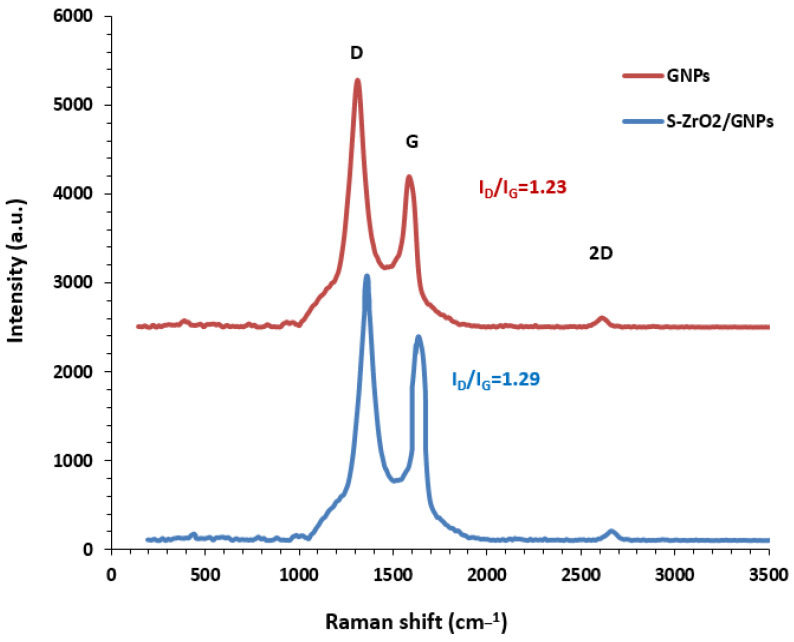
Raman characterization of GNP and S-ZrO_2_-GNP support.

The study was conducted to identify the vibrational modes of the GNPs and ZrO_2_-GNPs after sulfation. The G-band is a distinctive feature of the graphitic layers and represents the tangential vibration of sp2-bonded carbon atoms in a two-dimensional hexagonal lattice. Another characteristic feature of carbon materials is the presence of the D-band, which corresponds to the vibration of defective graphitic structures resulting from the doubly resonant disorder-induced mode [52,53,54,55,56,57,58,59,60]. The I_D_/I_G_ intensity ratio is a measurement of the quality of a sample. A higher value of this ratio indicates a more ordered structure. In Figure 3, the Raman spectra for the GNPs and S-ZrO_2_-GNPs are shown. The G (graphite) and D (disorder) bands and their second-order harmonic (2D band) are visible in the spectra at 1585.66 cm^−1^, 1311.30 cm^−1^, and 2611.92 cm^−1^ in the GNPs, respectively. The form of these bands is not impacted by doping with S-ZrO_2_, indicating that the overall structure of the graphene sheet remains intact during sulfation treatments or doping. Moreover, the Raman spectra of the two nanostructures presented in Figure 3 show that the intensity ratio of D-band to G-band (I_D_/I_G_) does not significantly change with the addition of zirconia. However, the peaks of D-, G-, and 2D-band have shifted to slightly higher wavelengths. This indicates that the addition of zirconia through the adopted method does not introduce specific functional groups to the surface of the GNPs [61,62].

The FTIR results of GO and ZrO_2_-graphene nanoplates and sulfated zirconia-GNPs were presented in our previously published work [63].

#### 2.1.4. XRD Pattern Characterization

**Figure 4:**
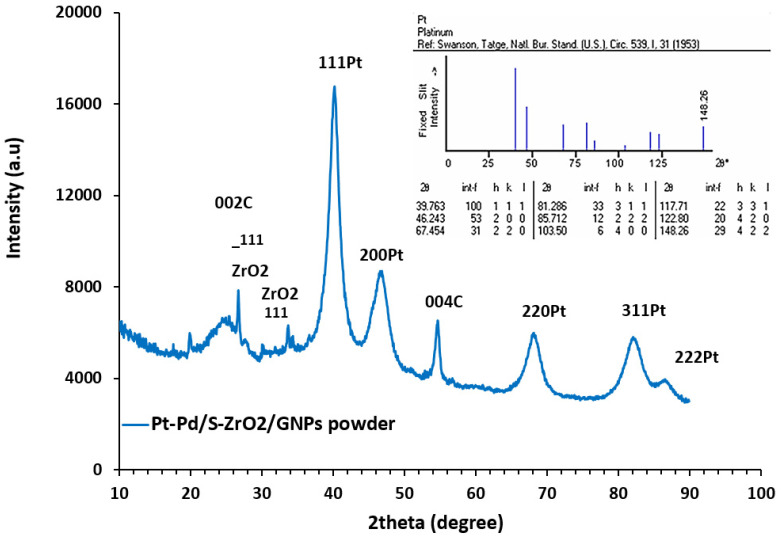
XRD diffraction chart of Pt-Pd/S-ZrO_2_-GNP nanocomposite along with standard PDF card of Pt [71].

The crystal lattice formation of the Pt-Pd/S-ZrO_2_-GNP electrocatalyst is shown in Figure 4, indicating the presence of carbon and platinum. The obtained nanocomposite exhibits the characteristic prominent peaks of the Pt Fcc structure at the Bragg angles of 86.44°, 82.08°, 68.05°, 46.62°, and 40.10° which relate to the (222), (311), (220), (200), and (111) diffraction peaks of the crystal surface, respectively, showing that it is a disordered Pt-Pd alloy. The formation of the bimetallic Pt-Pd nanoparticles in the Pt-Pd/S-ZrO_2_-GNP electrocatalyst was confirmed by a small transference of the XRD spectrum to greater angles due to the reduction in the lattice compared to Pt, indicating that the interatomic space of platinum was reduced by the replacement of the smaller Pd atom in the Pt metal lattice. The sharper diffraction peaks at 2θ = 26.651° and 54.27° are the features of the parallel GNP layers in the Pt-Pd/S-ZrO_2_-GNP composite, signifying a highly graphitic organization of the GNP in faces of (002) and (004) correspondingly. These plans show that the S-ZrO_2_ does not affect the graphitic structure of the GNPs. The preservation of the graphitic structure of the carbon is advantageous in the preparation of an electrocatalyst because it maintains the conductivity of the support materials. Besides the characteristic peaks of the graphite and Pt construction, some additional peaks at 2θ of 29.95° and 31.69° with the surfaces of −111 and 111 [64,65,66,67,68,69] were found in the nanocomposite, which is related to the ZrO_2_ nanocrystals used in the composition of the catalyst. The average size of the Pt-Pd nanoparticles was evaluated from the Debby–Scherrer formulation using the full width at half maximum (FWHM) of the (111) plane. This reflection was chosen for the Scherrer examination due to its high-strength level. This formula can be defined as follows [70]:

(1) $$d=0.9\frac{\lambda }{\mathrm{Bcos}\theta }\;\;$$

where d is the diameter of the mean particle size in $\overset{\cdot }{A}$; λ is the X-ray wavelength (1.5406 $\overset{\cdot }{A}$) for Cu${K\;}_{\alpha }$; θ is the Bragg angle; and B is the FWHM in radians. Based on the sample’s Pt(111) reflection, the average sizes of the Pt particles were calculated to be 4.50 nm with a d-spacing of 0.2246 nm (2.22 $\overset{\cdot }{A}$). The XRD results indicated that the innate attributes of the catalyst were not altered by the treatment of the crystalline ZrO_2_ with H_2_SO_4_.

#### 2.1.5. Chemical Composition of Synthesized Nanocomposite

**Figure 5:**
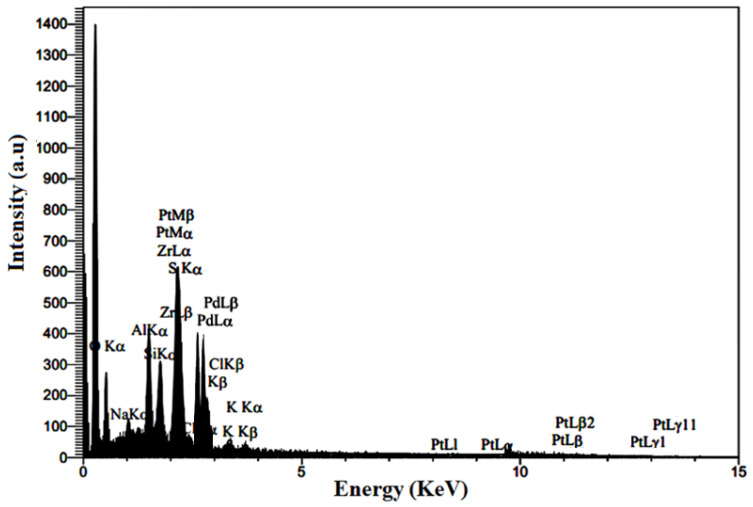
EDX pattern of Pt-Pd/S-ZrO_2_-GNP electrocatalyst.

Figure 5 illustrates the composition of the Pt-Pd/S-ZrO_2_-GNP electrocatalyst. The main ingredients of the spectrum are Pt, Pd, Zr, S, and C (from the GNPs). The sulfur comes from sulfated zirconia. Additionally, the ingredients Si, Al, and K were present. The solid silicon and Al peaks are likely due to the Si and Al substratum used in the FESEM examination. A small quantity of potassium observed in the spectrum may be caused by the KMnO_4_ used in graphene oxide production. A relatively trivial amount of Na and CL observed in the EDS image is essentially from the NaCl electrolyte used in the plating bath. Table 1 provides further information on the chemical composition of the synthesized nanocomposite resulting from the energy-dispersive X-ray (EDX) spectrum. The Pt-Pd (with the atomic ratio of 0.68%/0.61%) loading on the electrocatalyst containing the graphene nanoplates was quantitatively computed as 18.24%, which is close to the theoretic extent of 20 wt%.

### 2.2. Electrochemical Measurements

#### 2.2.1. Electrochemical Surface Area (ECSA) of Electrodes

**Figure 6:**
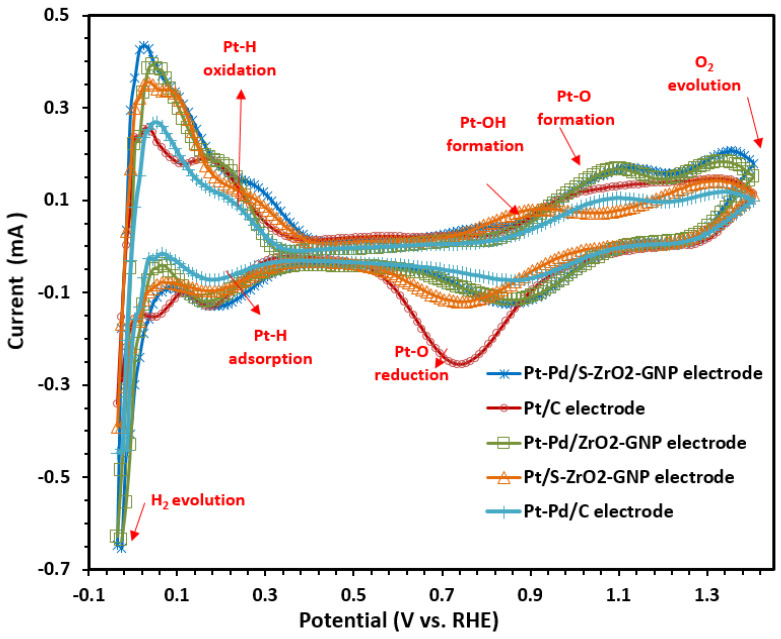
CVs of Pt-Pd/S-ZrO_2_-GNPs, Pt-Pd/ZrO_2_-GNPs, Pt/S-ZrO_2_-GNPs, Pt-Pd/C, and Pt/C in 0.1 M ${\mathrm{HClO}}_{4}$ solution scan rate: 50${\;\mathrm{mVs}}^{-1}$ at 25 °C under N_2_ flux.

Cyclic voltammetry is a commonly used electroanalytical technique to study electroactive species and electrode surfaces. It is usually the first electrochemical study performed. This technique is used to measure the ECSA of electrocatalysts using the hydrogen desorption method in a three-electrode system [72]. In Figure 6, the steady-state cyclic voltammograms of the different working electrodes, such as Pt-Pd/S-ZrO_2_-GNPs, Pt-Pd/ZrO_2_-GNPs, Pt/S-ZrO_2_-GNPs, and Pt/C, are presented. These electrodes were submerged in a 0.1 M ${\mathrm{HClO}}_{4}$ solution saturated by 99.9995% N_2_ in the voltage range between −0.2 and 1.2 V vs. Ag/AgCl (saturated KCl) at the scan rate of 50 mVs^−1^. All given potentials were converted and reported in the reversible hydrogen electrode (RHE) scale using the following: E_RHE_ = E_Ag_/_Agcl_ + 0.205 V (=$E_{\mathrm{RHE}}=E_{\mathrm{Ag}/\mathrm{AgCl}}+\emptyset_{\mathrm{Ag}/\mathrm{AgCl}}+0.0591\left(V\right)\times \mathrm{pH})$ [73,74].

The figure shows clear hydrogen absorption/desorption peaks ranging from 0.04 to 0.3 V vs. RHE, along with distinct surface oxidation and reduction peaks. The electrochemical surface area (ECSA) of the electrocatalysts was evaluated by computing the hydrogen adsorption/desorption area after double-layer correction, using a conversion factor of 210 µCcm^−2^ for the polycrystalline Pt. The ECSA (m^2^ g_metal_^−1^) was calculated as follows [75,76]:

(2) $$\mathrm{ECSA}\left(m^{2}{g_{\mathrm{metal}}}^{-1}\right)=\lceil \frac{Q_{H-\mathrm{adsorption}}\left(C\right)}{210{\mu \mathrm{Ccm}}^{-2}\times L_{\mathrm{Pt}}\left({\mathrm{mgcm}}^{-2}\right)\times A_{s}\left({\mathrm{cm}}^{-2}\right)}\rceil 10^{5}$$

where L_Pt_ is the Pt loading (mg cm^−2^) on the surface of an electrode, Q_H_ is the hydrogen adsorption charge (mCcm^−2^), and $A_{s}$ is the electrode surface area.

The comparison of the cyclic voltammograms of the Pt-Pd/S-ZrO_2_-GNP, Pt-Pd/ZrO_2_-GNP, Pt/S-ZrO_2_-GNP, Pt-Pd/C and commercial Pt/C electrodes in Figure 6 indicates that the double-layer of the Pt-Pd/S-ZrO_2_-GNP electrode is a little thinner than that of the Pt/S-ZrO_2_-GNP electrode due to the contribution of Pd. Additionally, the current density of the H_2_ release area extends when Pd is added, indicating a variation in the ECSA value of the catalyst due to Pd addition. Furthermore, adding Pd to platinum caused a slightly positive shift in the start of the anode O_2_ chemisorption (oxide development) and the reduction peak voltage of the oxide. For instance, the start of the anode oxygen chemisorption for the Pt-Pd/S-ZrO_2_-GNP, Pt-Pd/ZrO_2_-GNP, and Pt-Pd/C electrodes occurred at approximately 0.87 V, 0.83 V, and 0.82 (vs. RHE), in comparison to 0.79 V for Pt/C (this value for Pt/S-ZrO_2_-GNPs was 0.75 V). The contrast in the O_2_ chemisorption and oxide formation between the Pt/S-ZrO_2_-GNPs and Pt/C relative to the Pt-Pd/S-ZrO_2_-GNP, Pt-Pd/ZrO_2_-GNP, and Pt-Pd/C electrodes is due to the transition metal Pd. It suggests that the addition of Pd (alloying process) hinders the chemisorption of OH on the platinum sites at high voltages by altering the electronic effects (increase Pt d-band vacancy) and geometric (decrease in the Pt-Pt bond distance) factors [77,78]. This might facilitate the O_2_ adsorption at low overpotential and, therefore, enhance the oxygen reduction reaction kinetic performance. Also, the cathodic current peaks associated with the reduction of platinum oxide positively shift for the Pt-Pd/S-ZrO_2_-GNP, Pt-Pd/ZrO_2_-GNP, and Pt-Pd/C electrodes as compared to the Pt/S-ZrO_2_-GNP and Pt/C electrodes. This implies that the desorption of the oxygenate species (e.g., OH) from the surfaces of the alloy particles is easier than from the surface of pure Pt, i.e., the oxygenate species have lower adsorption energy on the Pt-Pd alloy catalysts. Since the adsorption of OH or other oxygenate species on the platinum surface can inhibit its catalytic activity toward oxygen reduction reaction, the weak adsorption of the oxygenated species would increase the surface active sites for ORR. Table 2 lists the ECSA for the Pt-Pd/S-ZrO_2_-GNP, Pt-Pd/ZrO_2_-GNP, Pt/S-ZrO_2_-GNP, Pt-Pd/C, and Pt/C electrodes. According to the table, the ECSA of Pt-Pd/S-ZrO_2_-GNPs is more significant than that of Pt/C, indicating that a more significant quantity of active sites is available for H_2_ adsorption and desorption reactions. This could be due to the structural modifications caused by alloying, the high specific surface area (SSA) of GNPs as catalyst support as well as the positive effects of ZrO_2_ as a promoter. Moreover, comparing the ECSA value of the Pt-Pd/S-ZrO_2_-GNPs, Pt-Pd/ZrO_2_-GNPs show that the catalyst layer containing sulfated ZrO_2_-GNP support utilizes a significant amount of Pt, suggesting that the sulfating increases the efficiency of PEMFCs. Furthermore, from Table 2, it is seen that the bimetallic Pt-Pd/C electrode had a relatively lower Pt surface area than the monometallic Pt/C electrode. This was expected because some Pt surface atoms are covered by Pd atoms. The smaller ECSA value of the Pt-Pd/C electrode in comparison to the Pt/C electrode also can be explained by the obtained XRD results. The reduced Pt active surface area of the Pt-Pd/C electrode is consistent with its higher particle size produced by alloying.

It can be seen that the Pt-Pd/S-ZrO_2_-GNP electrode had a higher Pt-based metal active surface area (97.32 m^2^ · g^−1^metal^−1^) compared to the Pt/C electrode (68.83 m^2^ · g^-1^Pt^-1^). The combination of sulfation, alloying, and GNPs helped maintain the catalytic activity of the Pt-Pd/S-ZrO_2_-GNP composite.

#### 2.2.2. Mechanism of Reaction

The activity of the Pt catalyst towards oxygen reduction is improved by the presence of a Pd alloy structure on S-ZrO_2_-treated GNPs. It is proposed that the presence of S-ZrO_2_ in the catalyst promotes the activity of ORR by PtPd which is explained by the flowing reactions [79,80].

(3) $$\mathrm{Pt}+O_{2}\to \mathrm{Pt}-O_{2}$$

(4) $$\mathrm{Pt}-O_{2}+{\;H}^{+}+e^{-}\to \mathrm{Pt}-{\mathrm{HO}}_{2_{\mathrm{ad}}}$$

(5) $$\mathrm{Pt}+\mathrm{Pt}-{\mathrm{HO}}_{2}\to \mathrm{Pt}-\mathrm{HO}+\mathrm{Pt}-O$$

(6) $${\mathrm{ZrO}}_{2}+H_{2}O\to {\mathrm{ZrO}}_{2}-{\mathrm{OH}}_{\mathrm{ad}}+{\;H}^{+}+e^{-}$$

(7) $$\mathrm{Pt}-O+{\mathrm{ZrO}}_{2}-{\mathrm{OH}}_{\mathrm{ad}}+3H^{+}+3e^{-}\to \mathrm{Pt}+{\mathrm{ZrO}}_{2}+2H_{2}O$$

(8) $$\mathrm{Pt}-\mathrm{HO}+{\mathrm{ZrO}}_{2}-{\mathrm{OH}}_{\mathrm{ad}}+2H^{+}+2e^{-}\to \mathrm{Pt}+{\mathrm{ZrO}}_{2}+2H_{2}O$$

It is well known that a catalyst for oxygen reduction needs to break the O-O bonds of oxygen and facilitate the reaction of the ORR. During this reaction, some formed intermediates like OH and O species are strongly adsorbed on the Pt surface. The surface oxygen of S-ZrO_2_ helps reduce the adsorbed HO and/or O on the Pt surface [81]. So, the enhancement of the Pt-Pd/S-ZrO_2_-GNP activity towards ORR is likely to occur at the interface of Pt and S-ZrO_2_ in the reactions (7) and (8). The importance of the surface oxygen source for the effective O_2_ reduction in fuel cell electrocatalysts has been highlighted in the case of carbon-coated tungsten oxides [82]. ZrO_2_ is a well-known reducible oxide that shows high oxygen storage capacity and strong metal support interaction between Pt and ZrO_2_ [63,83,84,85]. The overall reaction mechanism for oxygen reduction on the Pt–Pd/S-ZrO_2_-GNP electrode can be proposed as follows:

(9) $$\mathrm{PtPd}\ldots S-{\mathrm{ZrO}}_{2-2x}\left(\mathrm{interface}\right)+2{\mathrm{xO}}_{2}+4{\mathrm{xH}}^{+}+4{\mathrm{xe}}^{-}\to \mathrm{PtPd}\ldots S-{\mathrm{ZrO}}_{2}\left(\mathrm{interface}\right)+2{\mathrm{xH}}_{2}O$$

The enhancement of oxygen reduction is likely to occur at the interface of PtPd and S-ZrO_2_ in the supported system. In the presence of S-ZrO_2_ as a promoter, O_2_ adsorption starts at a higher potential at the interface of PtPd… S-ZrO_2_ thereby decreasing the anodic overpotentials. The Pt–O and Pt-H species are reduced to H_2_O at the PtPd… S-ZrO_2_ interface [81].

#### 2.2.3. ORR Kinetics

The prepared electrodes were evaluated in two ways. Firstly, the kinematic current and the number of exchanged electrons were calculated in the experiments conducted on the rotating disk electrode (RDE). Secondly, the mass transfer corrected Tafel plots constructed from the RDE data and the kinetic parameters of i_0_ were determined through the linear sweep voltammetry (LSV) analysis.

##### Estimation of Electrocatalyst Efficiency Using the RDE Instrument

**Figure 7:**
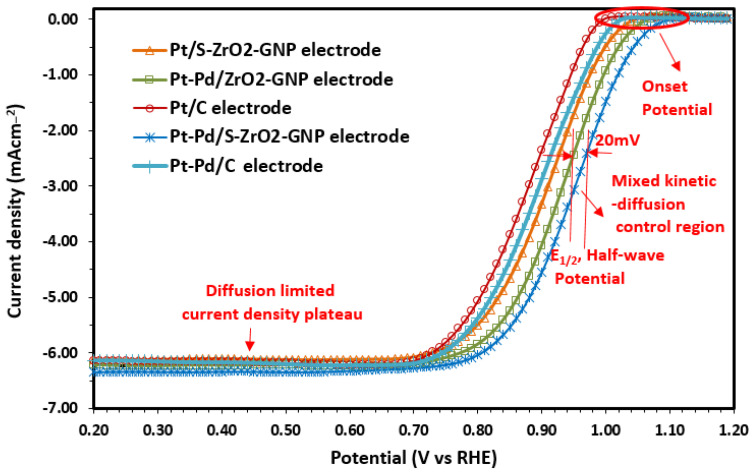
Polarization curves for ORR on Pt-Pd/S-ZrO_2_-GNP, Pt-Pd/ZrO_2_-GNP, Pt/S-ZrO_2_-GNP, Pt-Pd/C, and Pt/C electrodes in $0.1{\;M\;\mathrm{HClO}}_{4}$ solution scan rate: 5${\;\mathrm{mV}\;s}^{-1}$ rotation rate: 1600 rpm.

In the present study, the RDE method was used to investigate the oxygen reduction reaction (ORR) of the five types of electrodes Pt/C, Pt-Pd/C, Pt/S-ZrO_2_-GNPs, Pt-Pd/ZrO_2_-GNPs, and Pt-Pd/S-ZrO_2_-GNPs. The electrodes were prepared and tested in 0.1 M HClO_4_ at room temperature in a three-electrode cell. The ORR experiments were examined by flowing 99.999% O_2_ at a rate of 100 mL/min through the cell for 30 min using an MFC (model ALIGAT, SCIENTIFIC SCinTIFic, Tucson, AZ 85743, USA). The scanning voltage ranged from 1.1 to 0 V versus RHE, with a scan rate of 5 mV/s at a rotation speed of 1600 rpm. The results (Figure 7) showed that the onset potential of the Pt-Pd/S-ZrO_2_-GNPs was shifted by 30 mV, 50 mV, 70 mV, and 90 mV to a positive direction when compared to the Pt-Pd/ZrO_2_-GNPs, Pt/ZrO_2_-GNPs, Pt-Pd/C, and Pt/C, respectively (from 1.01 for Pt/C to 1.1 V for Pt-Pd/S-ZrO_2_-GNPs). It was observed that the electrochemical reaction occurred under mixed control, which was a combination of kinetic and diffusion control, ranging from 1.1 V to 0.7 V (vs. RHE). The diffusion limiting currents were achieved in the potential region under 0.7 V. The half-wave potential (E_1/2_, the potential where the current is half of its limiting value, the point of half-way between zero current and the diffusion-limited current density plateau) of the Pt-Pd/S-ZrO_2_-GNPs was found to be 960 mV, which was higher than that of the Pt/C, Pt-Pd/C, Pt/ZrO_2_-GNPs, and Pt-Pd/ZrO_2_-GNPs, which were 890 mV, 900 mV, 910 mV, and 940 mV, respectively. This indicates that the Pt-Pd/S-ZrO_2_-GNP catalyst has higher activity and is more promising than the other catalysts for PEMFC cathode applications [74,76,86].

The loading amount of Pt and ECSA was normalized to obtain the mass and specific activities, correspondingly. The current values of mass and specific activity at 0.9 V (where influences of mass transport are negligible) for the Pt-Pd/S-ZrO2-GNPs were found to be as high as 45.43 mA mgPt^−1^ and 0.0466 mA mgPt^−1^, respectively. In contrast, the values for Pt-Pd/ZrO_2_-GNP, Pt/S-ZrO_2_-GNP, Pt-Pd/C, and Pt/C electrodes were 40.75 mA mgPt^−1^ and 0.0431 mA mgPt^−1^; 33.29 mA mgPt^−1^ and 0.0401 mA mgPt^−1^; 28.67 mA mgPt^−1^ and 0.0402 mA mgPt^−1^; and 23.54 mA mgPt^−1^ and 0.0342 mA mgPt^−1^, respectively. The improved mass activity of the Pt-Pd/S-ZrO_2_-GNPs can be attributed to the bifunctional mechanism of the Pt-Pd alloy nanoparticles, as well as the interface effect between the Pt-Pd nanoparticles and O_2_ vacancy-rich ZrO_2_ nanoparticles and GNPs.

**Figure 8:**
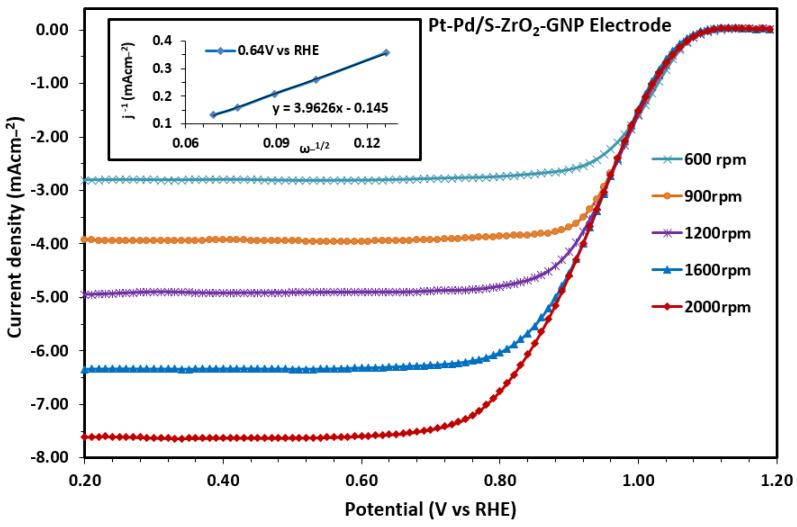
Disk current density achieved for the duration of the ORR in the cathodic scanning from the Pt-Pd/S-ZrO_2_-GNP electrode at various RDE rotation speeds (scan rate: 5 ${\mathrm{mV}\;s}^{-1}$). The inset displays the Koutechy–Levich plot attained at the diffusion flat terrain at 0.64 V.

The hydrodynamic behavior of the Pt-Pd/S-ZrO_2_-GNP electrode in the ORR was studied through rotating disk electrode experiments at different rotation speeds in an oxygen-saturated 0.1 M HClO_4_ solution at a scan rate of 5 ${\mathrm{mV}\;s}^{-1}$. The results of the experiments are presented in Figure 8. The ORR current was measured using the Koutechy–Levich equation, a standard metric for comparing various electrocatalysts [74,76,87,88].

(10) $$\frac{1}{I}=\frac{1}{I_{k}}+\frac{1}{{B\omega }^{\frac{1}{2}}}$$

(11) $$B=0.62{\mathrm{nFD}}^{\frac{2}{3}}\vartheta^{\frac{-1}{6}}C_{O_{2}}$$

where D, ϑ, and C are the diffusion coefficient, kinematic viscosity, and dissolved oxygen concentration, respectively, in a 0.1 M HCLO_4_ solution [89]. ω is the rotation speed in radians, and F is Faraday’s constant. $I_{k}$ and B are defined as the kinematic current and Levich parameter, respectively.

The calculations were performed using Equations (10) and (11), with the following values: D_O2_ = 1.9 × 10^−5^ cm^2^ s^−1^, ϑ = 9.87 × 10^−3^ cm^2^ s^−1^, and C_O2_ = 1.6 × 10^−6^ mol cm^−3^. The Faraday constant is 96,485 C mol^−1^. The calculated number of exchanged electrons with the kinematic current value of 3.13 mAcm^−2^ is 3.95 (n ≈ 4), which is in accordance with most scientific publications [90,91,92,93]. Consequently, oxygen reduction proceeds via the overall 4-electron path on the Pt surface of the Pt-Pd/S-ZrO_2_-GNP electrode. A similar result was also obtained from the intercept of the inset curve of Figure 8. The inset in Figure 8 shows the current region for the Pt-Pd/S-ZrO_2_-GNP catalyst.

##### Determination of Kinetic Parameters of b and $i_{0}$

The kinetic parameters were obtained after the measured currents were corrected for diffusion to give the kinetic currents in the mixed activation–diffusion region calculated based on the following equation:

(12) $$i_{k}=\frac{i\times i_{d}}{i_{d}-i}$$

where $\frac{i_{d}}{i_{d}-i}$ is the mass transfer correction.

**Figure 9:**
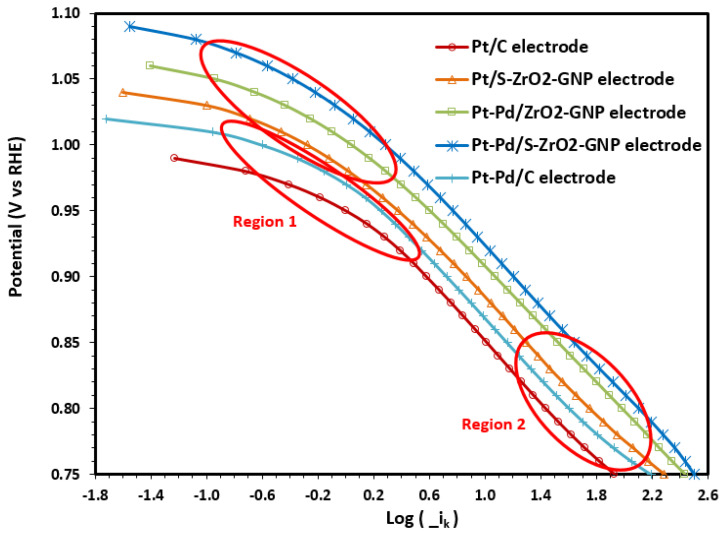
Mass transfer corrected Tafel plots for the ORR on Pt-Pd/S-ZrO_2_-GNP, Pt-Pd/ZrO_2_-GNP, Pt/S-ZrO_2_-GNP, Pt-Pd/C, and Pt/C electrodes obtained from the RDE rotation speeds of 1600 rpm (sweep rate: 5 ${\mathrm{mV}\;s}^{-1}$) in an O_2_-saturated solution of 0.1 M HClO_4_.

The Tafel plots (Figure 9) for Pt-Pd/S-ZrO_2_-GNPs, Pt-Pd/ZrO_2_-GNPs, Pt/ZrO_2_-GNPs, Pt-Pd/C, and Pt/C were examined by plotting log(−i_k_) against overpotential. According to the Tafel equation, the relation between the electrode overpotential (η) and current density is specified as follows [94,95]:

(13) $$\eta =-\frac{2.3\mathrm{RT}}{n\left(1-\alpha \right)F}{\mathrm{logi}}_{0}+\frac{2.3\mathrm{RT}}{n\left(1-\alpha \right)F}\log(-i_{k})$$

where η = (E − $E^{0}$) is the difference between the applied voltage and the Nerst voltage, R is gas constant, T is absolute temperature, n is the number of exchanged electrons, α is the transfer coefficient, F is Faraday’s constant, and $i_{k}$ and $i_{0}$ are the kinetic current density and exchange current density, respectively [95,96]. The equation can be simplified as follows:

(14) $$\eta =a+\mathrm{blog}\left(-i_{k}\right)$$

where $b=\frac{2.3\mathrm{RT}}{n\left(1-\alpha \right)F}$ and is called Tafel slope and $a=-\frac{2.3\mathrm{RT}}{n\left(1-\alpha \right)F}{\;\mathrm{logi}}_{0}$. This equation implies that in a certain current density range, overpotential is linearly dependent on the logarithm of current density. The exchange current density can be obtained from the intercept (at η=0) at the current density axis. In the higher Tafel slope, the kinetics of the ORR reaction is lower [96].

In Figure 9, the mass-transfer corrected Tafel plots from the RDE data are presented. Even though it is clear that the Tafel slope for the ORR is changing continuously on the potential range examined, in this figure, two Tafel regions with characteristic slopes near −120 and −60 mVdec^−1^ are distinguished, a transition in slope occurring at potentials between 0.95 and 1.02 V (vs. RHE, from 0.95 for Pt/C to 1.02 V for Pt-Pd/S-ZrO_2_-GNPs). The value of −120 mVdec^−1^ indicates that the rate-determining step is the transfer of the first electron to the O_2_ molecule. As suggested previously, the change in the slope is not related to the change in the reaction mechanism, but it has been attributed to the potential-dependent coverage of the surface oxides that inhibit the adsorption of oxygen molecules and reaction intermediates [97,98,99,100].

The experimental data were fitted to the two Tafel slope regions at low (E > 0.9 V, region1) and high overpotentials (E < 0.85 V, region2). The kinetic parameters were obtained using the Tafel equation and are presented in Table 3. They are in good agreement with the Tafel slopes for the carbon-supported Pt catalysts [88,101,102,103], with values around $-\frac{2.3\mathrm{RT}}{n\left(1-\alpha \right)F}$ at low overpotentials and values of ca. $-2\times \frac{2.3\mathrm{RT}}{n\left(1-\alpha \right)F}$ at high overpotentials.

Based on Figure 9, it is clear that the Pt-Pd/S-ZrO_2_-GNP electrode by itself offers a substantial improvement over the Pt/C electrode. Obvious from this figure is the higher activity for ORR for the Pt-Pd/C electrode than the monometallic Pt/C electrode. The other Pt-Pd/S-ZrO_2_-GNPs and Pt-Pd/ZrO_2_-GNPs are very similar in activity as can be seen in Figure 9. This can be concluded also from the Tafel slopes obtained at the two electrodes of Pt-Pd/S-ZrO_2_-GNPs and Pt-Pd/ZrO_2_-GNPs in Table 3. This suggests that the same rate-determining step is occurring at two electrodes. The results in Figure 9 and Table 3 showed that the kinetic current was higher in the Pt-Pd/S-ZrO_2_-GNPs compared to the Pt-Pd/ZrO_2_-GNPs, Pt/ZrO_2_-GNPs, Pt-Pd/C, and Pt/C. This indicates that charge transfer is faster on the Pt-Pd/S-ZrO_2_-GNPs than on the others. These results are compatible with data reported in the references [73,104,105,106,107] for other Pt-alloy components. The improvement in the catalytic properties of Pt-alloys is attributed to various factors, such as the structural alterations caused by alloying, an increase in Pt d-band vacancy due to electronic factors, which weakens the strength of the metal-O band, and a reduction in the Pt-Pt bond space due to geometric factors [77,78]. GNPs are considered a favorable supporting material for enhancing the catalytic efficiency of Pt-based materials in fuel cell electrodes. Additionally, the use of sulfated metal oxides like sulfated zirconia as a Co-catalyst with Pt helps to enhance the electron and proton conductivity of S-ZrO_2_-GNP nanocomposites.

#### 2.2.4. Electrochemical Impedance Spectroscopy (EIS) Studies of the Electrodes

**Figure 10:**
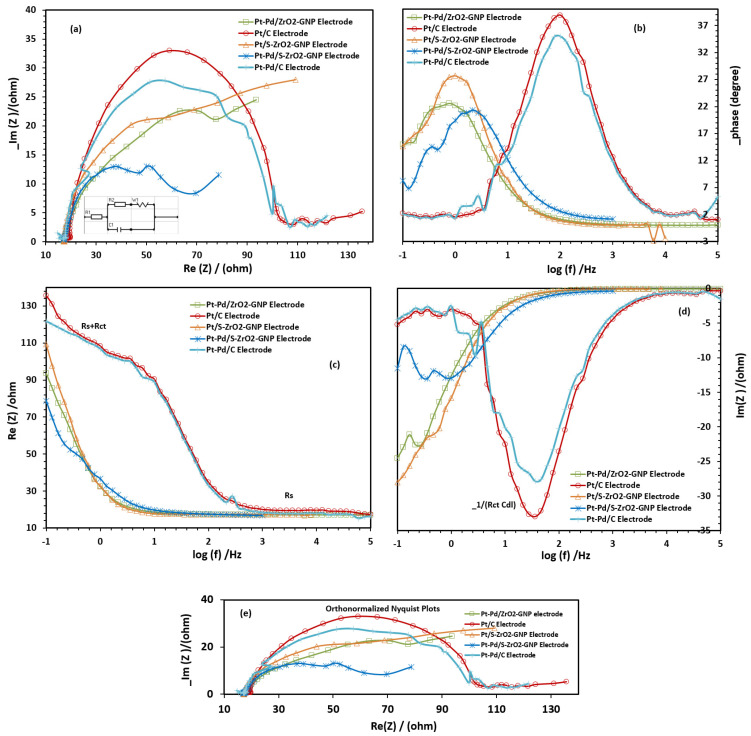
EIS chart in (**a**) Nyquist form, (**b**) bode phase plots, (**c**,**d**) bode magnitude diagrams, and (**e**) orthonormalized form of Nyquist plots of Pt-Pd/S-ZrO_2_-GNP, Pt-Pd/ZrO_2_-GNP, Pt/S-ZrO_2_-GNP, Pt-Pd/C, and Pt/C electrodes in 0.1 M HClO_4_ at 25 °C under O_2_ flux.

To seek the influence of carbon corrosion on electrode resistance changes and learn the interface process of electrodes, EIS analysis is utilized. The EIS examination of the electrodes was conducted at 0.05 V versus SCE by scanning frequencies ranging from 0.1 to 100 KHz with 67 decades and a changing sinusoidal signal of 0.05 V peak-to-peak superimposed on the DC potential. During the examinations, the electrodes were kept in an oxygen-saturated aqueous solution of 0.1 M *HClO*_4_, and all EIS measurements were completed below 25 °C. The corresponding Nyquist plots (imaginary against actual impedance) are presented in Figure 10a. The impedance spectra of all the electrodes exhibit similar characteristics in the Nyquist plots, i.e., a broken-down semi-circle at the high-frequency section, whose diameter corresponds to the charge transfer resistance demonstrating the catalytic activity for ORR, and relatively straight lines, approximately 45° (Warburg) in the low-frequency area which relates to the diffusion-limiting of the electrocatalyst (the impedance chart of the commercial Pt/C electrode displayed a half-circle at high-frequency region). In these plots, the actual axis intercept at the high-frequency region relates to the unadjusted resistance of the electrolyte solution in bulk. As can be seen, charge transfer and ohmic resistance (IR) increased harshly in the Pt-Pd/C and Pt/C after substantial carbon corrosion. However, no major fluctuations were seen for the Pt-Pd/S-ZrO_2_-GNP, Pt-Pd/ZrO_2_-GNP, and Pt/S-ZrO_2_-GNP samples (with sulfated zirconia-graphene nanoplate supports) suggesting simple oxygen reduction reactions on the surface of these electrodes. In fact, the charge transfer resistance (Rct) of the Pt-Pd/S-ZrO_2_-GNP, Pt-Pd/ZrO_2_-GN, and Pt/S-ZrO_2_-GNP electrodes is considerably lower than that of Pt-Pd/C and Pt/C electrodes due to the well-known conductivity of the GNPs, showing a high activity for ORR. The finest results were achieved for the Pt-Pd/S-ZrO_2_-GNP electrode, which exhibited a faster electron transfer and an improved mass diffusion kinetics after the sulfate treatment. Sulfated zirconia is responsible for less IR losses due to providing sufficient proton conductivity in the catalyst layer.

The contribution of each component to the cell resistance can be better understood by simplifying the circuits. In this experiment, the impedance data were fitted to appropriate equations, and the best equivalent circuits (EC) were selected, as displayed in the inset of Figure 10a. This circuit contains the sum of the electrode and electrolyte ohmic resistance (R1) with the charge transfer resistance (R2), which controls the electron transfer kinetics of electroactive kinds at the electrode boundary. The circuit also includes mass-transfer Warburge (W1) of the electrode parallel to the constant phase element, which belongs to the double-layer capacity (C1). In other words, the model R1 + (R2 + W1) × C1 corresponds to the CDC of R1(R2W1)C1.

In addition, the corresponding phase plots and bode diagrams in the real and imaginary parts of the impedance spectra of the studied electrodes are presented in Figure 10b–d and demonstrate that the ohmic resistance in all the electrodes did not change much during the ORR. However, in these diagrams, the lowest values of Rs+Rct and −1/Rct Cdl were found with the Pt-Pd/S-ZrO_2_-GNP electrode emphasizing that the O_2_ reduction reaction measured on the Pt-Pd/S-ZrO_2_-GNP electrode was significantly faster than the others. This may be an explanation for the higher performance in the Pt-Pd/S-ZrO_2_-GNP electrode in the polarization curve.

The orthogonal form of the Nyquist plots is also presented in Figure 10e.

#### 2.2.5. Long-Term Activity and Durability

**Figure 11:**
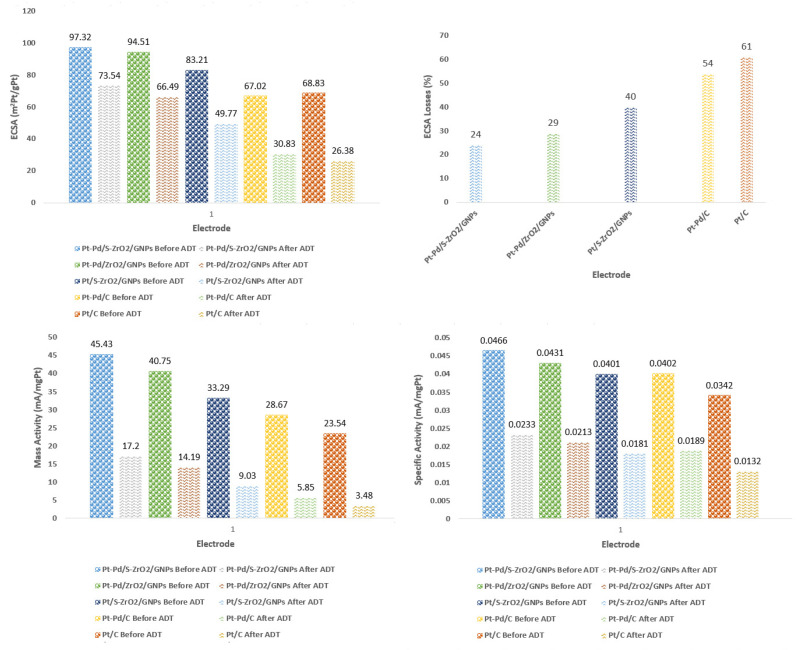
ECSA, mass, and specific activity based on RDE experiments at 0.9 V as well as ECSA values of Pt-Pd/S-ZrO_2_-GNP, Pt-Pd/ZrO_2_-GNP, Pt/S-ZrO_2_-GNP, Pt-Pd/C, and Pt/C electrodes before and after ADT test.

The effectiveness of the prepared electrocatalysts was evaluated through various indicators. The electrocatalysts were evaluated based on durability through successive voltage cycling in CV scanning and RDE. An accelerated durability test (ADT) was then performed on the four studied electrocatalysts. For CV scanning, first a working electrode with an electrodeposited catalyst in a plating bath covered with a Nafion-bonded catalyst layer dried in a vacuum oven at 80 °C (for the Pt/C electrode, the ink way prepared electrode, as described in Section 3.2.3, was used). The ADT involved cycling between potential −0.2 to 1.2 V (vs. Ag/AgCl reference electrode potential of 0.205 V stable in dilute acidic solution) for 500 cycles in a 0.1 M ${\mathrm{HClO}}_{4}$ solution saturated by high purity (99.9995%) nitrogen with a scan rate of 50 mV/s. Then, the ORR mass activity of the samples was examined in RDE configuration in an $O_{2}$-saturated $0.1{\;M\;\mathrm{HClO}}_{4}$ solution at a scan rate of 5${\;\mathrm{mV}\;s}^{-1}$ with a rotation speed of 1600 rpm. The corresponding mass and specific activity based on the RDE experiments, as well as the ECSA value after the ADT (also before the ADT), are presented in Table 4 and Figure 11.

**Figure 12:**
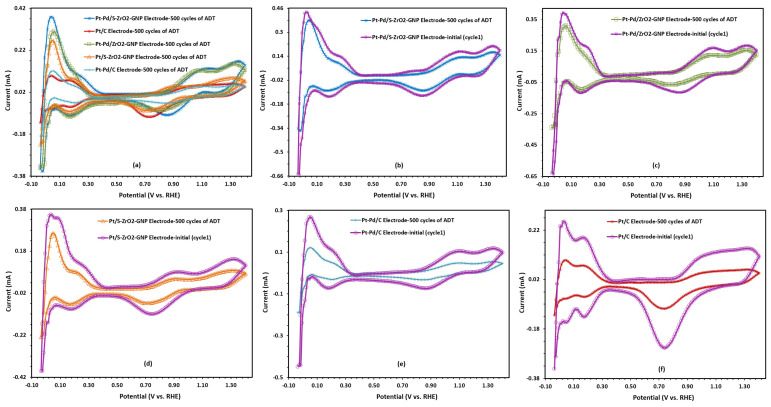
The CV curves (**a**–**f**) after 500 cycles of consecutive voltage scanning of the Pt-Pd/S-ZrO_2_-GNP, Pt-Pd/ZrO_2_-GNP, Pt/S-ZrO_2_-GNP, Pt-Pd/C, and Pt/C electrodes in a N_2_-saturated 0.1 M ${\mathrm{HClO}}_{4}$ solution, scan rate: 50 mVs^−1^.

The catalytic activity and stability of the Pt-Pd/S-ZrO_2_-GNPs, Pt-Pd/ZrO_2_-GNPs, Pt/S-ZrO_2_-GNPs, Pt-Pd/C, composite electrocatalysts, and Pt/C were evaluated from the ECSA measurement after the ADTs and the results are presented in Figure 12a–f. With cycling, a reduction in the H_UPD_ peak is observed for all electrocatalysts indicating an increase in the metal particle size. The loss of electrochemical surface area was observed on four catalysts during potential cycling caused by Pt dissolution and cluster formation. It is clear from Figure 12a that the ECSA for the Pt-Pd/S-ZrO_2_-GNP electrode is more significant than that of the Pt-Pd/ZrO_2_-GNPs, Pt/S-ZrO_2_-GNPs, Pt-Pd/C, and commercial Pt/C after the CV cycling, indicating both of the sulfating metal oxide/GNPs and alloying effects can preserve the electrocatalytic activity and stability of the composite. The ECSA values of the Pt-Pd/S-ZrO_2_-GNP electrode decreased by 24% after 500 cycles, while that of the Pt-Pd/ZrO_2_-GNP, Pt/S-ZrO_2_-GNP, Pt-Pd/C, and Pt/C electrodes decreased by 29, 40, 54 and 61%, respectively. The results disclosed that the Pt-Pd/S-ZrO_2_-GNP electrode was more electrochemically stable than the Pt-Pd/ZrO_2_-GNP, Pt/S-ZrO_2_-GNP, Pt-Pd/C, and Pt/C electrodes. Another important point that can be seen from Table 4 is that although the electrochemically active surface area of the bimetallic Pt-Pd/C electrode in the first state is lower than the monometallic Pt/C electrode, its ECSA value is higher than that of the Pt/C after the ADT tests, and this can be explained by the alloying effects, which prevent the increase in ECSA losses.

**Figure 13:**
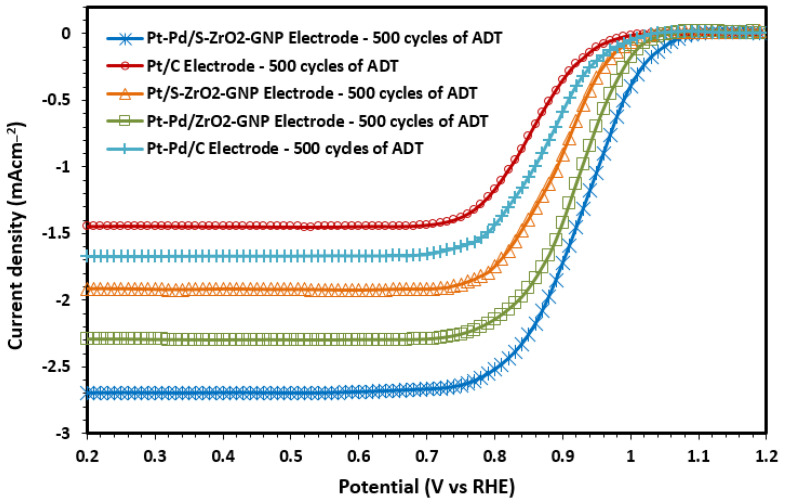
Polarization curves for ORR on Pt-Pd/S-ZrO_2_-GNP, Pt-Pd/ZrO_2_-GNP, Pt/S-ZrO_2_-GNP, Pt-Pd/C, and Pt/C electrodes after 500 cycles of ADTs in O_2_-saturated $0.1{\;M\;\mathrm{HClO}}_{4}$ solution, scan rate: 5 ${\mathrm{mV}\;s}^{-1}$ and rotation speed: 1600 rpm.

Also, the durability of each electrode was further examined by the RDE experiments in an $O_{2}$-saturated $0.1{M\;\mathrm{HClO}}_{4}$ solution at a scan rate of 5 ${\mathrm{mV}\;s}^{-1}$ with a rotation speed of 1600 rpm, and the results are presented in Figure 13. According to Table 4, the ORR mass activity of Pt-Pd/S-ZrO_2_-GNP composite electrocatalyst after 500 cycles at 0.9 V is 17.20 mA mg^−1^Pt^−1^, which is higher than that of the Pt-Pd/ZrO_2_-GNPs (14.19), Pt/S-ZrO_2_-GNPs (9.03), Pt-Pd/C (5.85), and Pt/C (3.48) after the ADTs. The results confirm once again that the Pt-Pd/S-ZrO_2_-GNP composite electrocatalyst has a high tolerance to electrochemical corrosion and premiere electrocatalytic performance in ORR.

## 3. Materials and Methods

### 3.1. Materials

Graphite powder (Gr) of 99.9999% purity was bought from Alfa Aesar, Heysham, Lancashire LA3 2XY, UK. Chemical materials, including H_2_PtCl_6_·6H_2_O (40%), ZrOCl_2_·8H_2_O, Na_2_NO_3_, KMnO_4_, H_2_SO_4_, 2-propanol, ethanol, (CH_2_OH)_2_, AgNO_3_, NaCL, and H_2_O_2_, were obtained from Sigma-Aldrich, Chemie GmbH, Eschenstr. 5, Taufkirchen, Germany. PdCl_2_ (59 wt.%) obtained from Scharlau chemie S.A., European Union, Pt/C (20 wt%) and Nafion^®^ solution (5 wt%, Dupont, Wilmington, Delaware) were purchased from Fuel Cell Earth Company, Woburn, MA, USA. O_2_ (99.999%) and N_2_ (99.9995%) gases were provided by Canadian Sigma Inc. 2149 Winston Park Drive, Oakville, ON L6H 6J8, Canada. Milli-Q water was utilized during the electrodeposition and electrochemical analysis. Polishing kits and glassy carbon (GC) electrode (d = 3 mm) were procured from Bio-Analytical System (BASi Corporate Headquarters, 2701 Kent Avenue. West Lafayette, IN 47906, USA).

### 3.2. Methods

#### 3.2.1. Synthesis of Graphene Nanoplates

To convert the pristine Gr to graphite oxide, the method of Hummer and Offenman was modified under a usual oxidation synthesis method [108]. In a container containing 90 mL of concentrated sulfuric acid (95%), 1 g of NaNO_3_ and 2 g of graphite were added to an ice bath. Gradually, 6 g KMnO_4_ was added and the mixture was agitated at 30 ± 5 °C for around 10 h. To the contents, 300 mL of deionized (DI) water was added and then (after about 30 min) diluted with 500 mL DI water. An amount of 5% H_2_O_2_ was added dropwise to the solution until the brown slurry turned yellow. The mixture was filtered, and the residue was dispersed in DI water through an ultrasonic system. By performing several centrifugation processes at 11,000 rpm for 25 min, the residue was washed out with 1:30 hydrochloric acid dilution and water to a pH value of 7. The filtrate was then dried in a vacuum furnace at 80 °C for 20 h. To generate graphene nanoplates, the resultant was dispersed in DI water and exfoliated through sonication via a welding horn. After decanting the mixture into a flask, NH_2_NH_2_. H_2_O (hydrazine monohydrate) was added as a reducing agent to the brown GO nanosheet dispersion. The solution was then refluxed at about 100 °C for 3 h which caused the color to repeatedly change into dark black due to the advent of the GNP dispersal floating on the surface of the solution. A small amount of the precipitate was removed through centrifugation for 10 min at 3500 rpm. The GNPs’ dispersion supernatants were directly dried in a vacuum oven to obtain the bulk of the graphene nanoplate powder.

#### 3.2.2. Synthesis of S-ZrO_2_-GNP Support

For synthesizing S-ZrO_2_-GNP nanocomposite, GNPs, ZrCl_2_ · 8H_2_O, H_2_SO_4_, and NH_3_ were used as the beginning material, sulfating agent, and precipitating agent. The blend was adjusted to a pH of 10 by regularly dripping 28% ammonia solution into zirconium oxychloride octahydrate solution (0.20 M) with proper amounts of GNPs and agitated for 36 h. The obtained ZrO_2_ · nH_2_O sol was washed with double distilled water by a centrifuge until chloride ions were not noticed by 0.1 N AgNO_3_, dried at 130 °C for 6 h, and powdered. The ZrO_2_ · 8H_2_O and GNP admixture was added to 0.50 M H_2_SO_4_ with strong stirring for 25 min, filtrated, and dried at 120 °C. Following that, the resulting powder was calcinated at 600 °C under airflow for 40 min, and the obtained support was regarded as S-ZrO_2_-GNPs. For comparison, the ZrO_2_-GNP powder was also prepared in the same way except that the sulfation step was not performed.

#### 3.2.3. Electrodeposition of Pt-Pd Nanoparticles on the S-ZrO_2_-GNP-Contained Glassy Carbon

A two-electrode cell was used to carry out the galvanostatic pulse co-electrodeposition of Pt-Pd. To begin, 10 mg of S-ZrO_2_-graphene powder was mixed with 1000 µL of isopropanol solution and sonicated for 30 min to create a soft slurry. Next, 10 µL of the slurry was micro-pipetted onto the top of the glassy carbon electrode and dried at 30 °C. The experiment used an S-ZrO_2_/graphene ink on glassy carbon as a cathode and Pt wire as an anode electrode. The electrodeposition process was carried out in a plating cell containing a solution of 4 mM H_2_PtCl_6_ · 6H_2_O + 4 mM PdCl_2_ dissolved in 0.5 M NaCl [109]. The pH of the solution was brought to 2.8 by adding HCl and NaOH. A peak current density of 300 mAcm^−2^ was applied with an on/off time of 10/100 ms, resulting in a duty cycle of 10% for the studied electrode. The solution was gently stirred during electrodeposition to pass new metal ions to the cathode and remove any gas bubbles produced. A total of 4 mM concentration of Pt and Pd metal precursors were calculated to reach 20% of the electrocatalyst value based on the theoretical assumption, and the step of applying the S-ZrO_2_/graphene slurry to the working electrode before electrodeposition was repeated to reach the support weight of 80% and was controlled by weighing. Before the Pt-Pd electrodeposition, the active surface of the glassy carbon containing sulfated-zirconia-doped graphene nanoplates was impregnated with a solution of 0.05% Nafion in ethanol. Since the S-ZrO_2_ nanocrystals act as a proton conductor in the catalyst layer, only a small amount of Nafion ionomer was required in the catalyst layer composition of the electrodes in comparison to the preparation of the conventional Pt/C electrodes. After the electrodeposition, the electrode was instantly taken away from the electrolyte, carefully washed with DI water, and dried out under an IR lamp. The total amount of charge passed through the electrode was 200 mCcm^−2^, corresponding to approximately 0.1 mgcm^−2^ of the Pt-Pd alloy catalyst according to a formula presented in our previously published article [51]. The amount of Pt-Pd deposited increased with an increase in charge passed. The metal deposition time, assuming a faradic yield of 100% is approximately 36,630 ms (37 s) with t_off_ = 100 ms and t_on_ = 10 ms.

**Figure 14:**
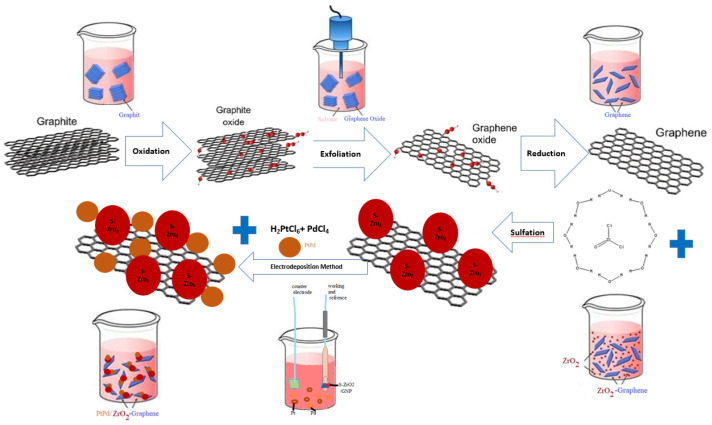
Schematic of experimental methodology for synthesizing S-ZrO_2_-GNPs and fabricating Pt-Pd/S-ZrO_2_-GNPs.

**Figure 15:**
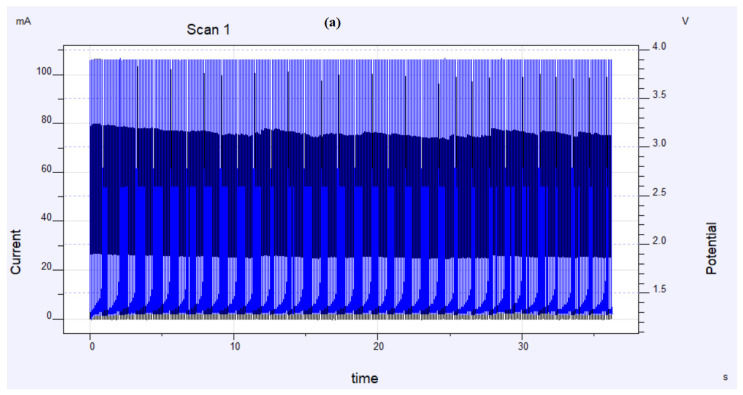

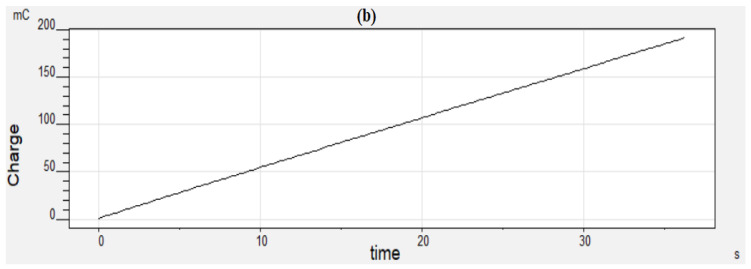
Presentation of the (**a**) current(potential)–time charts of the Pt-Pd/S-ZrO_2_-GNP electrode under the electrodeposition condition at the end of the electrodeposition process and (**b**) charge–time chart of the amount of Pt-Pd electrodeposited on the S-ZrO_2_-GNPs.

The experimental methodology to synthesize the Pt-Pd/S-ZrO_2_-GNP electrode is depicted in Figure 14. Also, the current(potential)–time charts of Pt-Pd/S-ZrO_2_-GNP electrode under the electrodeposition condition at the end of the electrodeposition process and the quantity of Pt-Pd electrodeposited on the GC surface have been shown in Figure 15a,b. A conventional Pt/C electrode was made up by spraying a slurry of commercial 20 wt% Pt/C nanoparticles in ink preparation way onto a GC electrode with a Pt/C loading of 0.1 mg/cm^2^ and an optimal Nafion load of 0.28 mg/cm^2^ according to the formula below which was introduced in ref. [110].

(15) $$\mathrm{Nafion}\;\left({\mathrm{mg}\;\mathrm{cm}}^{-2}\right)≅56\frac{L_{\mathrm{Pt}}}{P_{\mathrm{Pt}}}$$

where $L_{\mathrm{Pt}}$ is the platinum loading $\left({\mathrm{mg}\;\mathrm{cm}}^{-2}\right)$ and $P_{\mathrm{Pt}}$ the weight percentage of the metal supported on carbon (Pt/C).

#### 3.2.4. Characterization and Analysis

The Pt-Pd/S-ZrO_2_-GNPs were analyzed using an Equinox 3000 spectrometer (IENL France, Head Quarters, INEL, Paris, France). The analysis employed Cu Kα λ = 0.15406 nm radiation yielded at 40 kV and 30 mA with a resolution of ≤0.1°. The AFM (model Nanosurf Easyscan2, Nanosurf AG, Gräubernstrasse 12, 4410 Liestal, Switzerland) was used in contact mode to study the physical structure of graphene nanosheets. A field emission scanning electron microscopy (FESEM) depiction and EDX spectroscopy joined with SEM MAG100.00kx with a silicon detector were performed at 15 kV. The particle morphology and size of Pt-Pd nanoparticles deposited on the support are characterized by transition electron microscopy (TEM,EM208,1–100 kV, Philips, Eindhoven, The Neterlands). The FTIR spectra of GO, ZrO_2_/GNPs, and S-ZrO_2_-GNP composites were obtained using a WQF-510A/520 FTIR spectrometer (No.160 Beiqing Road, Haidian District, Beijing 100095, China). The Pt-Pd electrodeposition and electrochemical measurements as well as the ORR experiments were conducted on a conventional 2- and 3-electrode cell using Iviumstat potentiostat/galvanostat (Vertex, De Zaale 11, 5612 AJ Eindhoven, The Netherlands), respectively. A Pt foil was used as a counter electrode, Ag/AgCl saturated KCl as the reference electrode, and a glassy carbon disk as the working electrode. Due to safety considerations in the laboratory, instead of the Pt/H_2_/H^+^ standard dynamic hydrogen electrode, the most popular Ag/AgCl/Cl^−^ reference electrode was used to record data in an acidic solution of 0.1 M HClO_4_. Before use, the electrode surface was polished via alumina suspension with successively reduced particle sizes between 1 and 0.05 µm on polishing mats. The electrode was then ultrasonicated in C_2_H_5_OH and DI water for 15 min to eliminate contamination.

## 4. Conclusions

The corrosion of carbon supports and the need for more durable materials have led to the exploration of alloy- and ceramic-based support materials for Pt catalysts. Non-precious metal oxides are promising options due to their resistance to corrosion in severe fuel cell environments. However, their low surface area and electric conductivity prevent them from being used as primary support materials. In this paper, Pt-Pd/S-ZrO_2_-GNP nanocomposite electrode was prepared and compared with Pt-Pd/ZrO_2_-GNP, Pt/S-ZrO_2_-GNP, Pt-Pd/C, and Pt/C electrodes in terms of the electrochemical activity and durability for ORR using CV, RDE, polarization curves (Tafel plots) and EIS in acidic solutions. All the results showed that the Pt-Pd/S-ZrO_2_/GNPs gave a higher catalytic activity and durability for ORR. The Pt-Pd/ZrO_2_-GNP electrode had a higher electrochemical surface area with a positive shift peak potential for ORR than other studied electrodes. The ECSA of the Pt-Pd/S-ZrO_2_/GNPs was 97.32 m^2^/gPt, which was higher than that of the Pt-Pd/ZrO_2_-GNPs, Pt/S-ZrO2-GNPs, Pt-Pd/C, and Pt/C with values of 94.51, 83.21, 67.02, and 68.83 m^2^/gPt, respectively. Furthermore, the mass activity of the Pt-Pd/S-ZrO_2_/GNPs for ORR at 0. 9 V versus RHE was 45.43 mA mg^−1^Pt^−1^, which was 1.92 times higher than commercial Pt/C (23.54 mA mg^−1^Pt^−1^) based on RDE experiments. The electrocatalyst also demonstrated high activity for ORR cathode operation after 500 cycles of durability testing. The electrochemical surface area of the Pt-Pd/S-ZrO_2_/GNP electrode remained at 76% of its initial value. The charge transfer resistance of the Pt-Pd/S-ZrO_2_/GNPs was smaller than that of the Pt-Pd/ZrO_2_-GNPs, Pt/S-ZrO_2_-GNPs, Pt-Pd/C, and Pt/C, indicating an increase in reaction kinetics. The enhanced mass activity and durability of the Pt-Pd/S-ZrO_2_/GNPs could be attributed to the synergistic effect between Pt-Pd alloy NPs, oxygen vacancy-rich ZrO_2_ NPs, sulfation effect, and high conductive GNPs. The function of each component in the Pt-based electrocatalyst was analyzed to determine their impact on electrochemical surface area, electron, and proton conductivity. It was suggested that a collaborative effect exists between metal oxides (ZrO_2_), deposited Pt-Pd NPs, and GNP sublayer, which improves the electron transfer rate. In addition, another effect related to the attractive feature of the S-ZrO_2_/GNP composite increases electron and proton conductivity. Based on the results of this paper, the Pt-Pd/S-ZrO_2_-GNPs can be selected as one of the best electrodes with excellent ORR for PEMFC application. The application of sulfated metals, i.e., S-ZrO_2_ as a Co-catalyst of Pt-Pd, seems to be a promising oxygen reduction cathode catalyst.

## Figures and Tables

**Table 1:** Chemical composition of Pt-Pd/S-ZrO_2_-GNP nanocomposite (quantitative values).

Element	Line	wt%
C	Kα	52.84
O	Kα	11.27
Na	Kα	0.95
Al	Kα	2.43
Si	Kα	1.77
S	Kα	10.30
Cl	Kα	0.28
K	Kα	0.57
Zr	Lα	1.35
Pt	Lα	10.03
Pd	Lα	8.21
		100

**Table 2:** Electrochemical surface area of the Pt-Pd/S-ZrO_2_-GNP, Pt-Pd/ZrO_2_-GNP, Pt/S-ZrO_2_-GNP, Pt-Pd/C, and Pt/C electrodes with a catalyst loading of 0.1 mgcm^−2^ in a N_2_-saturated solution of 0.1 M ${\mathrm{HClO}}_{4}$.

Electrode	Crystallite Size (XRD) (nm)	$Q_{H}(C$)	$\mathrm{ECSA}\;\left(\frac{m^{2}}{g\;metal}\right)$
Pt-Pd/S-ZrO_2_-GNPs	4.50	14.443$\;\times \;10^{-4}$	97.32 **
Pt-Pd/ZrO_2_-GNPs	4.54	14.011$\;\times \;10^{-4}$	94.51 **
Pt/S-ZrO_2_-GNPs	4.31	12.338$\;\times \;10^{-4}$	83.21 *
Pt-Pd/C	4.39	9.94$0\;\times \;10^{-4}$	67.02 **
Pt/C(20 wt%)	4.20	10.20$5\;\times \;10^{-4}$	68.83 *

* Per unit weight of Pt. ** per unit weight of metals including both Pt and Pd.

**Table 3:** Kinetic parameters of Pt-Pd/S-ZrO_2_-GNP, Pt-Pd/ZrO_2_-GN, Pt/S-ZrO_2_-GNPs, Pt-Pd/C, and Pt/C electrodes in O_2_-saturated solution of 0.1 M HClO_4_.

Electrode	Tafel Slope: b(mVdec) in (E > 0.9 V)	Tafel Slope: b(mVdec) in (E < 0.85 V)	i_0_(Acm2) For (E < 0.85 V)
Pt-Pd/S-ZrO_2_-GNPs	−56	−106	1.662$\;\times \;10^{-3}$
Pt-Pd/ZrO_2_-GNPs	−57	−107	1.610$\;\times \;10^{-3}$
Pt/S-ZrO_2_-GNPs	−59	−111	1.021$\;\times \;10^{-3}$
Pt-Pd/C Pt/C (20 wt%)	−58	−113	1.020$\;\times \;10^{-3}$
	−61	−122	1.018$\;\times \;10^{-3}$

**Table 4:** Mass and specific activity based on RDE experiments at 0. 9 V as well as ECSA values of Pt-Pd/S-ZrO_2_-GNP, Pt-Pd/ZrO_2_-GNP, Pt/S-ZrO_2_-GNP, Pt-Pd/C, and Pt/C electrodes before and after ADT test.

Test	Electrode	Mass Activity at 0. 9 V (vs. RHE) (mA/mg metal)	Specific Activity (mA/mg metal)	$\mathrm{ECSA}\;\left(\frac{m^{2}Pt}{gmetal}\right)$
Before ADT	Pt-Pd/S-ZrO2-GNPs	45.43	0.0466	97.32
	Pt-Pd/ZrO2-GNPs	40.75	0.0431	94.51
	Pt/S-ZrO2-GNPs	33.29	0.0401	83.21
	Pt-Pd/C	28.67	0.0402	67.02
	Pt/C	23.54	0.0342	68.83
After ADT	Pt-Pd/S-ZrO2-GNPs	17.20	0.0233	73.54
	Pt-Pd/ZrO2-GNPs	14.19	0.0213	66.49
	Pt/S-ZrO2-GNPs	9.03	0.0181	49.77
	Pt-Pd/C	5.85	0.0189	30.83
	Pt/C	3.48	0.0132	26.38

## Data Availability

Data is contained within the article.
